# Nitric Acid in the Treatment of Umbilical Hernia in Colts

**Published:** 1897-03

**Authors:** 


Nitric Acid in the Treatment of Umbilical Hernia in Colts. M. M. F. Peuch has treated four cases of umbilical hernia in colts with nitric acid, and has had good results in each case. There is little fear of protrusion of the bowel, and, as a rule, but one application of the acid is necessary; if it does not have the desired effect, a second application may be made ten or fifteen days afterward.

The method of operation is very simple. Take a piece of oakum, and after saturating it with pure nitric acid paint the tumor over its entire circumference, passing the oakum over it three or four times.—*Journ. de Méd. Vétérin. et de Zootechnie,* 1896.

